# Design of a novel lateral mass screw–plate system for the treatment of unstable atlas fractures: a finite element analysis

**DOI:** 10.1186/s13018-024-04582-6

**Published:** 2024-02-05

**Authors:** He-Gang Niu, Jing-Jing Zhang, Yi-Zhu Yan, Cheng-Kun Zhao, Kun Yang, Yin-Shun Zhang

**Affiliations:** Department of Orthopedics, The First Affiliated Hospital of Anhui Medical University, No.218 Jixi Road, Hefei, 230022 Anhui Province People’s Republic of China

**Keywords:** Atlas fractures, Open reduction and internal fixation (ORIF), Cervical spine, Biomechanics, Finite element analysis

## Abstract

**Background:**

Osteosynthesis of unstable atlas fractures preserves joint motion and therefore has a distinct advantage over a range of treatment procedures. To prevent the potential disadvantages associated with osteosynthesis, a new atlas lateral mass screw–plate (LMSP) system has been designed. However, the biomechanical role of using the LMSP system in atlas internal fixation is not known. The aim of this study was to compare the biomechanical stability of a new LMSP with traditional posterior screw and rod (PSR) fixation techniques on the occipitocervical junction (C0–C2) through finite element analysis.

**Methods:**

A nonlinear C0–C2 finite element model of the intact upper cervical spine was developed and validated. The unstable model using the PSR system was then compared with the model using the LMSP system for fixation. A vertical load of 40 N was applied to the C0 to simulate head weight, while a torque of 1.5 Nm was applied to the C0 to simulate flexion, extension, lateral bending, and axial rotation.

**Results:**

The range of motion of both systems was close to the intact model. Compared with the LMSP system model, the PSR system model increased flexion, extension, lateral bending, and axial rotation by 4.9%, 3.0%, 5.0%, and 29.5% in the C0–C1 segments, and 4.9%, 2.7%, 2.4%, and 22.6% in the C1–C2, respectively. In flexion, extension, and lateral bending motion, the LMSP system model exhibited similar stress to the PSR system model, while in axial rotation, the PSR system model exhibited higher stress.

**Conclusions:**

The findings of our study indicate that the two tested system models provide comparable stability. However, better stability was achieved during axial rotation with the LMSP system, and in this system, the maximum von Mises stress was less than that of the PSR one. As the atlantoaxial joint functions primarily as a rotational joint, the use of the LMSP system may provide a more stable environment for the joint that has become unstable due to fracture.

## Introduction

There is still no consensus on the optimal treatment of unstable atlas fractures [1–4]. In the past, skull traction, external fixation of the Halo-vest head frame, and other non-surgical treatment methods were mostly used, but the treatment time was long, the patients were difficult to tolerate, and the clinical results were poor [5, 6]. Therefore, for better fracture healing and restoration of stability in the occipitocervical junction, it has been suggested by a majority of surgeons that patients with unstable atlas fractures should undergo surgical treatment at an early stage [6–8].

The traditional surgical methods are mainly atlantoaxial fusion or occipitocervical fusion [9–12], but these fusion operations sacrifice the motor function of the upper cervical spine, especially the rotational function of the atlantoaxial joint [13]. To preserve the motor function of the atlantoaxial joint, various open reduction and internal fixation (ORIF) procedures for unstable atlas fractures have been studied and reported by many surgeons recently [14–16]. Clinical follow-up revealed that patients with atlas transverse ligament injury also showed no significant atlantoaxial instability after ORIF, with good clinical outcomes [17–20].

ORIF is an emerging surgical technique in recent years, and scholars have tried a variety of surgical approaches and methods. Currently, the surgical methods include anterior transoral screws and plate reduction and fixation [21, 22], posterior screws and plate or PSR reduction and fixation [23, 24], as well as anterior and posterior combined approach reduction and fixation [25]. Among these, PSR fixation is the most common one, typically involving the placement of polyaxial pedicle screws in the lateral masses of the atlas, connected by a titanium rod, and then repositioned under pressure. During the operation, the posterior arch fracture of atlas can be easily reduced under direct vision. By pressing between the screws of the lateral mass on both sides, the displaced lateral mass can be effectively displaced and reduced, making the procedure relatively simple as compared to the transoral approach, while avoiding infectious complications. However, due to tail swing during compression of the polyaxial screws, it is difficult to anatomically reposition the fracture of the anterior arch of the atlas, and complications such as excessive bleeding and nerve injury may occur [26]. He et al. [18] designed and applied a posterior screws and plate system to simplify the operation and reduced the internal fixation notch but still could not effectively solve the reduction problem of anterior arch fractures of the atlas.

**Fig. 1:**
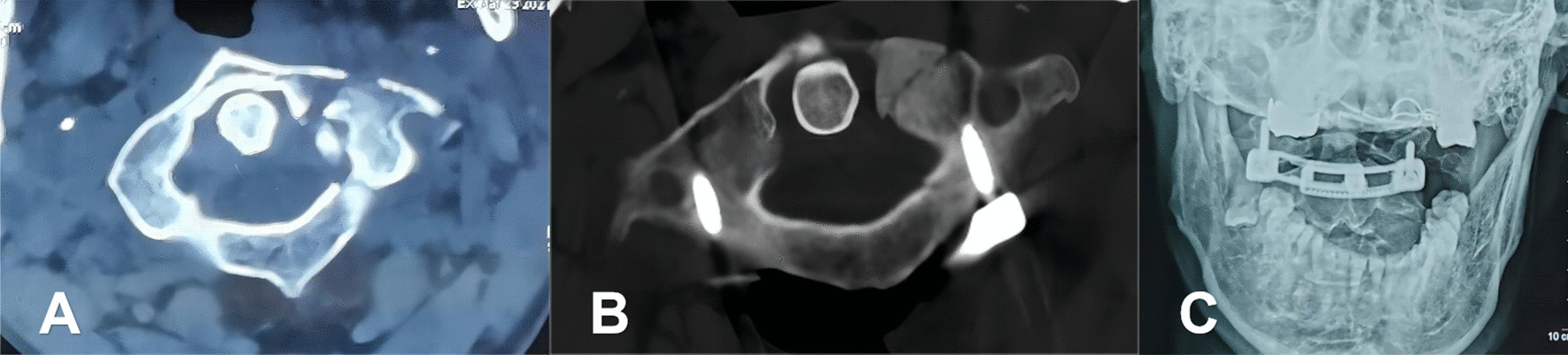
**A** A 55-year-old male presented with neck pain from a heavy object injury and a fracture of the anterior atlas arch on CT examination; **B** postoperative CT scans revealed satisfactory reduction; **C** postoperative open mouth X-ray image

To prevent the potential disadvantages associated with polyaxial screws and rod fixation, there is a need to identify a surgical method with a high degree of reduction and ease of operation. Our team had previously initiated a series of clinical studies for the treatment of unstable atlas fractures [27]. A novel atlas LMSP was used to treat unstable atlas fractures (Fig. 1). The study has shown that this new atlas LMSP can achieve better reduction results and easier operation. But compared to traditional, widely used PSR systems, does the new atlas LMSP have more biomechanical advantages? Current studies provide little information on the stability of different fixation devices for unstable atlas fractures. In this study, a new atlas LMSP system was designed using computer-aided techniques based on atlas imaging data. The aim of this study was to evaluate the biomechanics of two types of internal fixation using the range of motion (ROM) and stress distribution of the FEM at the C0–C2 segment.

## Materials and methods

In this study, four FEMs of the C0–C2 segments of the upper cervical spine were reconstructed. FEMs include the intact model, the unstable model, and the unstable model with the LMSP system or the PSR system implanted at C1 segment.

### Intact and unstable finite element model

The study was approved by the ethics committee of the First Affiliated Hospital of Anhui Medical University. A healthy male volunteer (age 24, height 181 cm, weight 75 kg) was selected, informed of the study content, and signed the corresponding informed consent form. A computed tomography (CT) scanning of the upper cervical spine was performed, and CT images of segments C0–C2 were obtained at 0.5-mm intervals. The images were imported into Mimics 19.0 (Materialise Company, Leuven, Belgium) in DICOM (Digital Imaging and Communications in Medicine) format for separation, erasure, filling, and other operations. The images were then imported into the Geomagic Wrap 2017 (Raindrop Company, Marble Hill, USA) in STL (Standard Template Library) format for smooth processing such as nail and redundant feature removal. Solidworks 2017 (Dassault Systemes S.A Company, Massachusetts, USA) was used to remove the overlapping parts of the cortical and cancellous bones and the curved entity, and the transverse ligament model was established. Finally, in ANSYS 17.0 finite element analysis software, the material property parameters of titanium alloy, cortical bone, cancellous bone, transverse ligament, and articular cartilage were established, springs were created, and spring stiffness was set to simulate the ligaments associated with the C0–C2 segment. Next, the model was divided into meshes. To ensure the accuracy of calculation to meet the requirements of analysis, the mesh type and mesh size were controlled, and the contact position mesh was refined. The mesh type was set as a 10-node tetrahedral mesh. In addition, ANSYS 17.0 can also analyze the stress distribution and ROM of the C0–C2 structure.

**Fig. 2:**
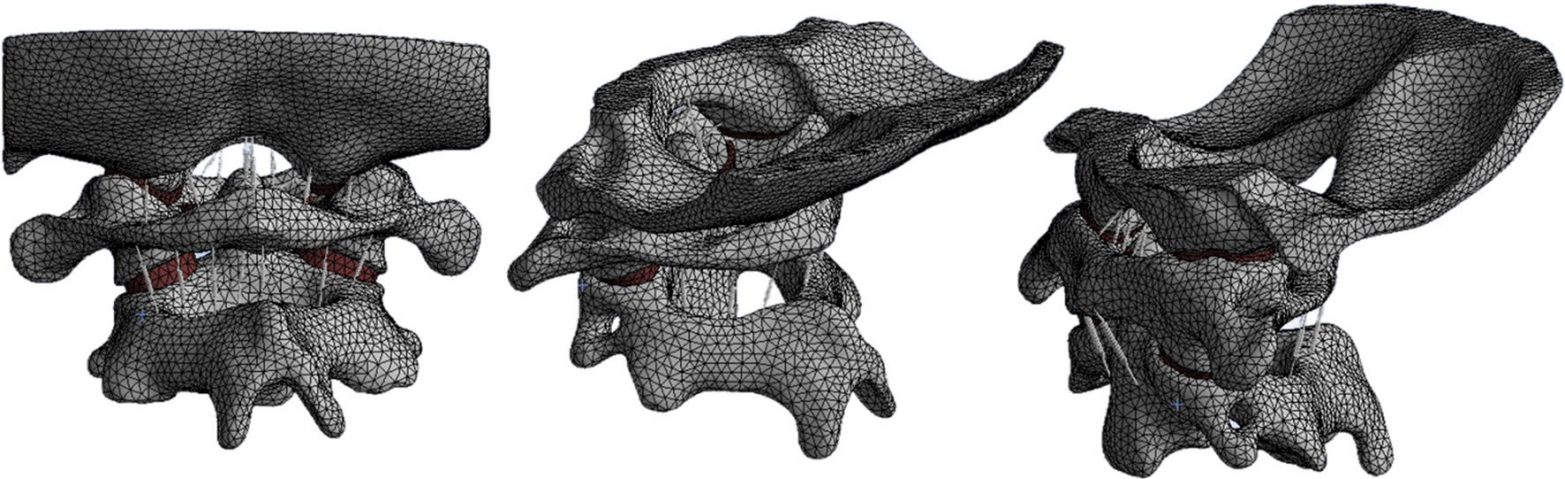
Finite element model of intact

Finally, the complete FEM consisted of the vertebral body and ligaments (Fig. 2). The vertebral body included cortical bone, cancellous bone, and articular cartilage. Ligaments include the transverse ligament, anterior atlanto-occipital membrane, posterior atlanto-occipital membrane, tectorial membrane, cruciate ligament-vertical portion, joint capsule, alar ligament, apical ligament, anterior longitudinal ligament, and posterior longitudinal ligament (Table 1). The transverse ligament is a low elastic tissue and is quite tough, so it was modeled with 4-node membrane elements [28, 29]. Sliding contact with friction was defined between the facet joints, the occipital condyle and the atlas, the atlas and the dens, the dens and the transverse ligament, and the atlas and the axis, with the coefficient of friction set to 0.1 [28, 30]. The material properties of the FEM were selected from previously published studies (Table 1) [28, 31–34].

**Table 1:** Material properties of upper cervical C0–C2 FEM

Components	Youngs modulus(MPa)	Poisson ratio	Element type
Cortical bone	12,000	0.29	C3D4
Cancellous bone	500	0.29	C3D4
articular cartilage	10	0.30	C3D4
LMSP and PSR	110,000	0.30	C3D4
Transverse ligament	20	0.30	4-node membrane elements
Anterior atlanto-occipital membrane	10	0.30	Spring element
Posterior atlanto-occipital membrane	10	0.30	Spring element
Tectorial membrane	10	0.30	Spring element
cruciate ligament-vertical portion	10	0.30	Spring element
joint capsule	10	0.30	Spring element
alar ligament	5	0.30	Spring element
apical ligament	10	0.30	Spring element
anterior longitudinal ligament	10	0.30	Spring element
posterior longitudinal ligament	10	0.30	Spring element

The most common type of unstable atlas fracture is Gehweiler type III, which is also known as the "Jefferson fracture" of the atlas [35]. Therefore, based on the established intact model, this study used the software's deletion element editing function to delete the transverse ligament of the atlas and move the lateral mass of the atlas outward at the maximum stress on both sides of the anterior and posterior arch, forming four fracture lines with a width of 2–3 mm, simulating the finite element analysis of four-parts of unstable atlas burst fracture with transverse ligament damage.

### Finite element model of atlas LMSP system

**Fig. 3:**
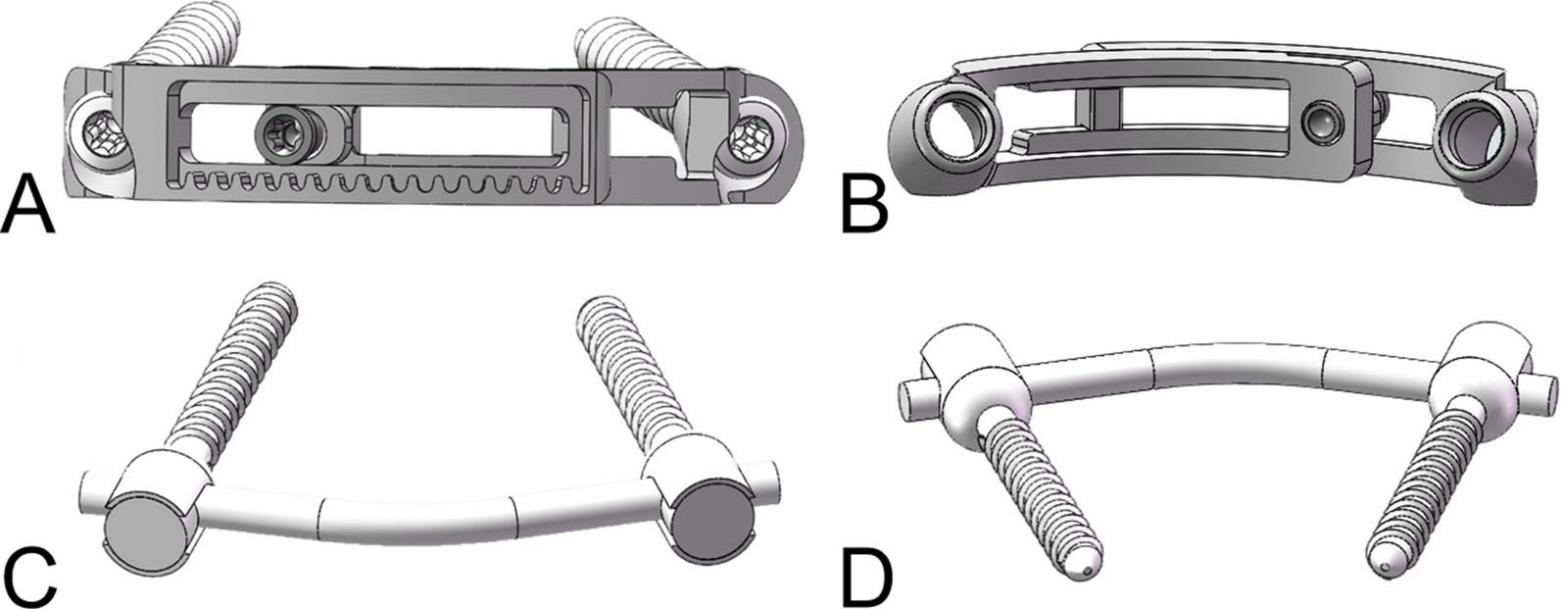
**A** The front view of LMSP system. **B** The rear view of the LMSP system. **C** The front view of PSR system. **D** The rear view of the PSR system

**Fig. 4:**
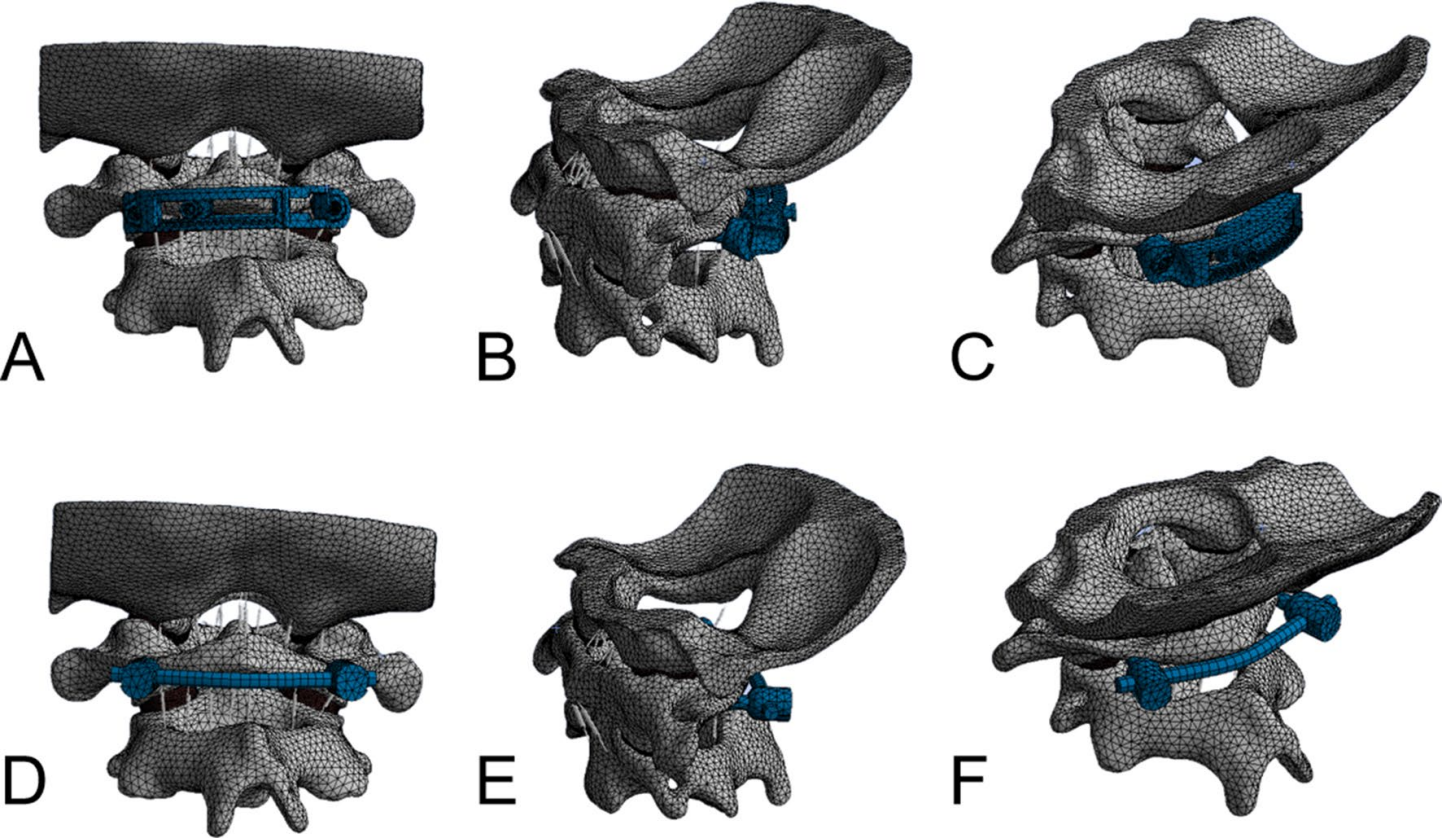
**A**–**C** Finite element model of atlas LMSP system **D–F** Finite element model of atlas PSR system

According to the anatomical characteristics of atlas and the operating process of the simple posterior surgical approach, a geometric model of the new posterior atlas LMSP system was constructed using Solidworks 2017 computer-aided software (Fig. 3). The new posterior atlas LMSP system consists of an upper curved fixing plate, a lower curved fixing plate, and two lateral mass screws. The two sides of the curved fixation plate are symmetrical in radians, with a radius of 26 mm and a plate thickness of 3 mm. A gear bar is provided on one side of the upper curved plate chute, and the lower arc plate is provided with a screw hole with adjustment nut in the screw hole so that the two curved fixing plates can be fixed and locked, and the relative position of the upper and lower curved fixing plates can be adjusted by rotating the adjusting nut to rotate the gear. The screw tails were designed to be tapered, and the upper and lower curved fixing plates were each provided with tapered screw holes that were angled inward and aligned with the screw tails (Fig. 3). A finite element model of the LMSP system for a new posterior atlas was created using finite element software such as ANSYS 17.0 for mesh generation, loading the titanium alloy material properties, and then setting the loads and boundary conditions (Fig. 4). The system model was imported into the unstable FEM. The embedded constraint commands from ANSYS were adopted to implement the screw and bone connections in FEM.

### Finite element model of atlas PSR system

PSR fixation has become arguably the most frequently used method for treating unstable fractures of the atlas in the past. Therefore, the FEM of the PSR system was established in this study and the operating procedures of the PSR have been described in detail elsewhere [17, 36]. The screw used in the experiment was a polyaxial screw with a diameter of 3.5 mm, a length of 26 mm, and a length of 140 mm for the connecting rod (Fig. 3). The rod was bent to approximate the curvature of the posterior arch of the atlas and fastened with polyaxial screws. After placing the screws and rod, all connections were fully tightened. Finally, they were imported into the unstable FEM to generate a FEM of the posterior PSR system (Fig. 4).

### Boundary and loading conditions

The same boundary and loading conditions were applied to both models. ANSYS 17.0 was used to constrain the mobility of the lower surface of the C2 vertebrae, limiting its ROM in six directions. A concentrated force of 40 N vertically downward was applied to the top of the occipital bone to simulate head weight, while a torque of 1.5 Nm was applied to the occipital bone to produce flexion, extension, lateral bending, and axial rotation of the models in all directions of motion [31]. The ROM of the C0–C2 segment and the MVMS of the implant were quantified. ROM and stress distributions of the new LMSP system fixed FEM and the PSR system fixed FEM were compared.

## Results

### Validation of FEM

To validate our model, we calculated the ROM of the C0–C1 and C1–C2 segments of the intact model and compared them with the in-vitro study by Panjabi et al. [37–39], as well as the findings of two FEM by Zheng et al. [40] and Zhang et al. [32] (Table 2). We found good agreement between the previous ROM data and our results. Furthermore, by deleting the transverse ligament and fracture line formation, the unstable model increased the ROM in flexion, extension, lateral bending, and axial rotation by 57.5%, 20.4%, 27.1%, and 58.7% in C0–C1 segments, and 25.5%, 29.5%, 143.6%, and 37.0% in C1–C2 segments, respectively, compared to the intact model (Table 3).

**Table 2:** Validation of FEM

Motion	Panjabi et al.[37]		Panjabi et al.[38, 39]		Zheng et al.[40]		Zhang et al.[32]		Intact Model	
	C0–C1	C1–C2	C0–C1	C1–C2	C0–C1	C1–C2	C0–C1	C1–C2	C0–C1	C1–C2
Flexion	3.5 ± 0.6	11.5 ± 2.0	10.8–17.2	9.8–16.2	4.6	11.7	14.5	15.0	4.0	9.8
Extension	21.9 ± 1.9	10.9 ± 1.1	10.8–17.2	6.0–16.0	20.7	9.5	13.3	12.7	22.6	10.5
Lateral bending	5.6 ± 0.7	4.0 ± 0.8	2.6–8.6	3.8–19.6	6.6	4.7	5.5	5.9	5.9	3.9
Axial rotation	7.9 ± 0.6	38.3 ± 1.7	1.0–10.5	24.2–46.4	7.1	39.1	8.5	30.6	7.5	37.3

**Table 3:** ROM of C0–C2 segments under different loading conditions

Motion	Intact model		Unstable model		LMSP		PSR	
	C0–C1	C1–C2	C0–C1	C1–C2	C0–C1	C1–C2	C0–C1	C1–C2
Flexion	4.0	9.8	6.3	12.3	4.1	10.2	4.3	10.7
Extension	22.6	10.5	27.2	13.6	23.1	11.0	23.8	11.3
Lateral bending	5.9	3.9	7.5	9.5	6.0	4.1	6.3	4.2
Axial rotation	7.5	37.3	11.9	51.1	7.8	38.5	10.1	47.2

### ROM at the C0–C2 level

The changes in ROM in flexion, extension, lateral bending, and axial rotation of the two internal fixation models were compared under the same load (Table 3). Both the LMSP system and the PSR system significantly reduced ROM as compared to the unstable state. Compared with the intact state, the PSR system model increased ROM in flexion, extension, lateral bending, and axial rotation by 7.5%, 5.3%, 6.8%, 34.7%, and 9.2%, 7.6%, 7.7%, 26.5% for C0–C1 and C1–C2 segments, respectively. Compared with the intact state, the LMSP system model showed increases in flexion, extension, lateral bending, and axial rotation of 2.5%, 2.2%, 1.7%, 4.0%, and 4.1%, 4.8%, 5.1%, 3.2% ROM for C0–C1 and C1–C2 segments, respectively, indicating that both models were stable under normal physiological loading. Compared with the LMSP system model, the PSR system model increased ROM by 4.9%, 3.0%, 5.0%, 29.5%, and 4.9%, 2.7%, 2.4%, and 22.6% for flexion, extension, lateral bending, and axial rotation at C0–C1 and C1–C2 segments, respectively. This indicates that the LMSP system may provide similar stability in terms of flexion, extension, and lateral bending, but may provide higher stability in terms of axial rotation as compared to the PSR system.

### Stress distribution on the implants

**Fig. 5:**
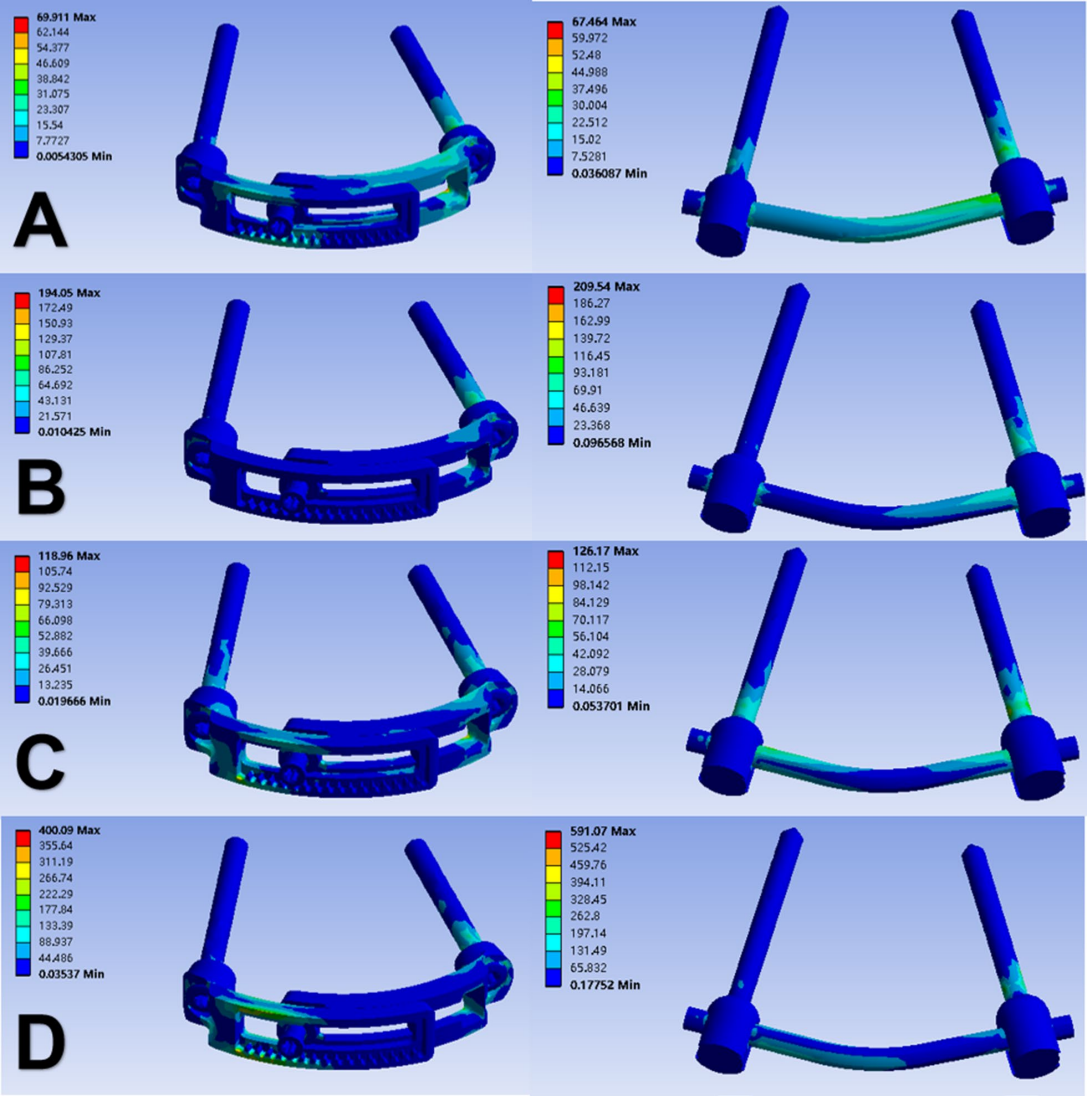
von Mises stress contour plots of the LMSP and PSR systems in the state of equilibrium under different loading conditions after applying a vertical load of 40 N: **A** flexion, **B** extension, **C** lateral bending, **D** axial rotation

**Fig. 6:**
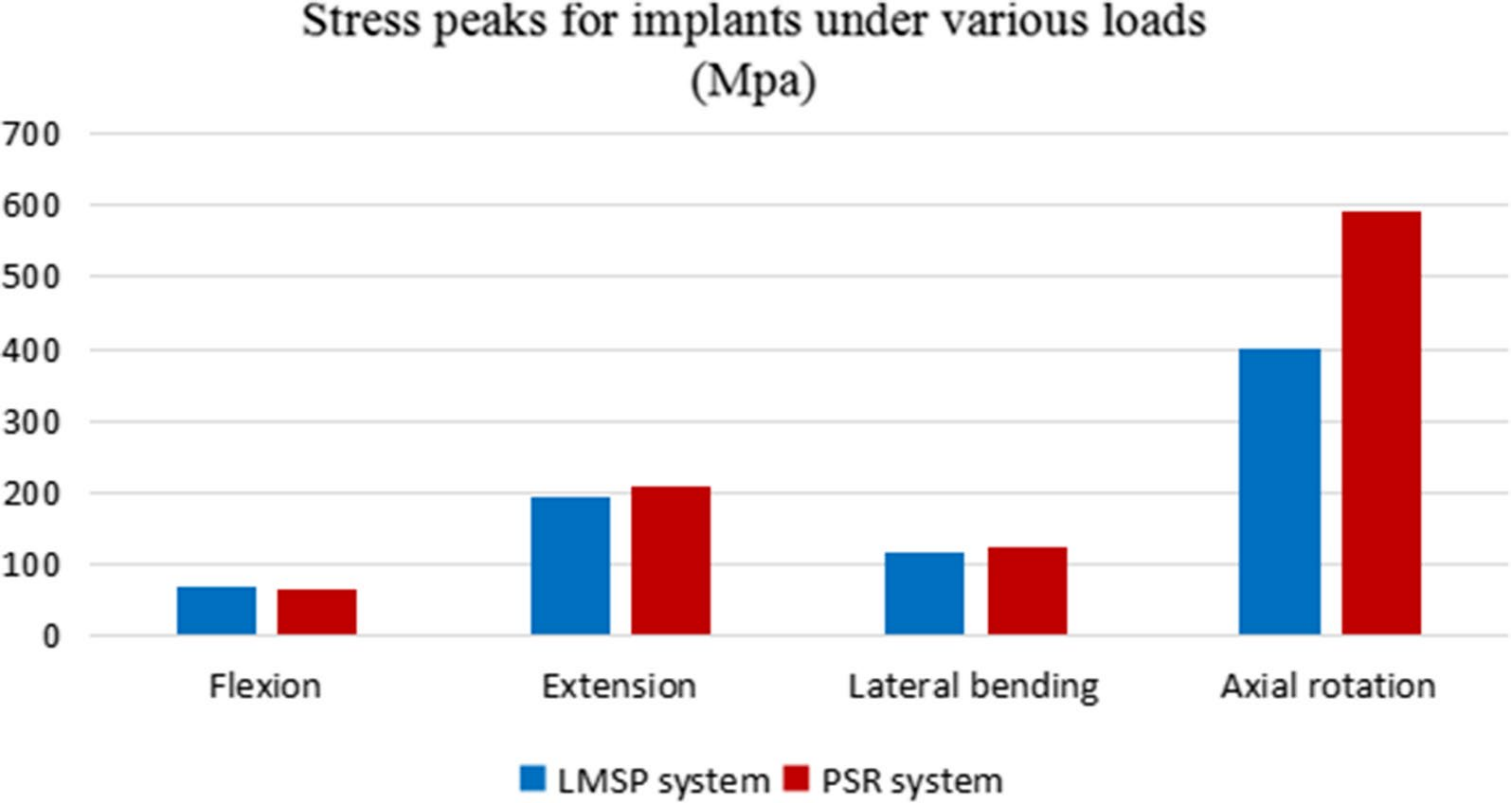
Comparison of stress peaks for implants

The von Mises stress contour plots demonstrate that the stress distribution regions are comparable for each fixation technique (Fig. 5). Different loads were applied to the fixation system from four different directions. The MVMS on the LMSP system was determined to be 69.91 MPa in flexion, 194.05 MPa in extension, 118.96 MPa in lateral bending, and 400.09 MPa in axial rotation. The comparison shows that the MVMS of the PSR system was 67.46 MPa in flexion, 209.54 MPa in extension, 126.17 MPa in lateral bending, and 591.07 MPa in axial rotation. The MVMS ratios for the LMSP system and the PSR system were 1:0.96 in flexion, 1:1.08 in extension, 1:1.06 in lateral bending, and 1:1.48 in axial rotation, respectively. The results showed that the LMSP system had similar MVMS to the PSR system in flexion, extension, and lateral bending but significantly lower MVMS in axial rotation (Fig. 6).

The concentration of MVMS in the LMSP system mainly occurred at the junction of the screw and the curved plate. The concentration of MVMS in the PSR system mainly occurred at the junction of screws and bones and at the connecting rods. Both the LMSP and PSR systems had the highest peak stress in axial rotation and the lowest peak stress in flexion.

## Discussion

Early unstable atlas fractures are mainly treated with non-surgical methods, such as skull traction, neck brace, and Halo-vest head frame external fixation. However, the atlas fracture is poorly reduced and problems such as non-union and displacement of the fracture may occur. At the same time, non-surgical treatment may take a longer time, which may lead to patient intolerance and related complications, eventually requiring surgical treatment. In 2005, Dvorak et al. [6] conducted a study on the long-term quality of life in patients with atlas burst fractures. The majority of patients received non-surgical treatment, and the results showed that it was difficult for patients to recover to pre-injury levels, indicating that non-surgical treatment is not the most ideal treatment method.

Conventional atlantoaxial or occipitocervical fusion for the treatment of unstable atlas fractures sacrifices upper cervical motor function and significantly reduces the quality of life after surgery. In recent years, to preserve the motor function of the atlantoaxial joint, various ORIF techniques for atlas fractures have been reported, and satisfactory clinical results have been achieved [17–19, 26, 36, 41]. Currently, the reported ORIF for atlas fractures treatment includes anterior transoral approach, posterior screw rod or plate reduction and internal fixation, as well as combined anterior and posterior approach internal fixation. Intraoperative reduction in atlas fractures is challenging due to the deep location and narrow space of the anterior transoral procedure. Although some scholars have improved the reduction effect by improving the surgical reduction and fixation plate [21, 22], postoperative infection is still a problem that should not be ignored. The posterior approach is generally used to implant polyaxial screws in lateral masses on both sides of the atlas and connect them with a titanium rod for compression reduction and fixation. During the operation, the fracture of the posterior arch of the atlas can be easily reduced under direct vision. However, when the fracture separation in the anterior arch is large, the reduction in the fracture line of the anterior arch is not ideal [19, 26], leading to prolonged fracture healing time or even non-union of the fracture. This may be related to a deviation in the direction of transmission of lateral compression reduction force through the posterior approach. Bohm et al. [25] added an anterior surgery based on posterior compression reduction and internal fixation of atlas fractures, using steel wires to bind and tighten the heads of the lateral mass screws that penetrate the anterior bone cortex, so that better reduction could be achieved. However, the surgical trauma is significant and difficult to operate.

In recent years, some scholars have used monoaxial screws not only to complete the reduction and fixation of atlas fractures through simple posterior surgery but also to drive the lateral mass to rotate forward and inward through the force generated by the anterior swing of the screw during the locking process to adapt to the curved titanium rod, thereby promoting effective reduction in atlas anterior arch fractures [7, 42]. However, when using a monoaxial screw rod system in the posterior approach, during the compression reduction operation, the long-arm sleeve needs to be placed at a large swing angle on both sides of the screw tail to achieve lever reduction. At the same time, the assistant needs to maintain pressure on both sides of the screw with compression pliers and also needs to prevent the connecting rod from rotating, which is difficult to operate and causes high stimulation of the paravertebral muscle.

In this study, we designed a new LMSP system by taking the advantages of monoaxial screws and combining it with the anatomical features of the atlas. Previously, through imaging measurements, we found that the distance between the posterior tubercle of the atlas and the posterior margin of the foramen magnum was significantly greater than the distance between the posterior tubercle of the atlas and the superior margin of the axial, which resulted in the impact of the connecting rod of the PSR system on the axial arch during the posterior extension of the cervical spine after the operation, affecting the patient's posterior extension movement. For this reason, we designed the tapered screw holes of the curved fixing plate to be inwardly inclined (Fig. 3), effectively avoiding the impact problem. By measuring the radius of the posterior atlas arch, the new LMSP system designed based on this data can be better adapted to the posterior atlas arch. In addition, we found that the anatomical structure of the atlas may be individualized in relation to gender and height. Therefore, we designed different models of new LMSP systems that can provide personalized treatment for patients.

The new atlas LMSP system consists of two adjustable curved fixing plates and two atlas lateral mass screws. The two curved fixing plates adjust the relative position by an adjusting nut, which not only makes the operation simple but also reduces the traction stimulation on the paravertebral muscles and enables compression reduction in posterior arch fractures of the atlas under direct vision. Compressive reduction in the anterior atlas arch fracture was achieved by matching the tapered screw holes of the curved plates to the tapered structure of the screw tails, producing the effect of anterior adduction of both screws. But the biomechanical properties of the new device remain unclear.

Current FEM studies of the upper cervical spine are mostly limited to the study of cervical fusion surgery, and few FEM studies of ORIF treatment of atlas fractures have been conducted. In this study, two finite element reconstruction models, the LMSP and the PSR systems were successfully developed by loading the internal fixation system model based on the Jefferson fracture model. Combining the same posterior fixation method with the same loads applied from four different directions, the results of our ROM study indicated that the ROM of the LMSP and PSR systems (Table 3) was close to intact model, both of which had the advantage of preserving upper cervical motion function. Compared with the LMSP system model, the PSR system model increased the flexion, extension, and lateral bending of C0–C1 and C1–C2 segments by 4.9%, 3.0%, and 5.0%, as well as 4.9%, 2.7%, and 2.4%, respectively, while axial rotation increased by 29.5% and 22.6%, respectively. This indicates that compared to the PSR system, the LMSP system has similar stability in flexion, extension, and lateral bending, but greater stability in axial rotation. These differences in ROM controls between the two devices may be caused by differences in the length and thickness of the curved fixing plate and the titanium rod. However, the research design was only a finite element study, and further biomechanical cadaver studies are needed to investigate the current findings.

The stress distribution on the implant is closely related to the long-term stability of the fixation technique. The von Mises stress contour plot showed that the stress in the LMSP system was mainly concentrated in the tapered screw holes area of the curved plates and the screw tails, while the stress in the PSR system was mainly concentrated in the interface area between the connecting rods and the screws, as the screw is required to transfer the mechanical load to the rear rod. Our data also indicate that during the initial loading to equilibrium state, the von Mises stress in the PSR system is greater than the stress in the LMSP system (except for flexion motion). Compared with the PSR system, the LMSP system exhibits similar high stress peaks on the implant in terms of flexion, extension, and lateral bending. During axial rotation, the LMSP system has a smaller MVMS than the PSR system. As the atlantoaxial joint functions primarily as a rotational joint, the use of the LMSP system can provide a more stable force environment for the joint that has become unstable due to fracture.

There are still several potential limitations to this study. Firstly, the finite element analysis model established in this study only included the C0–C2 segments of the upper cervical spine and did not include the other segments of the cervical spine, nor did it include the muscles and other soft tissues of the upper cervical spine. Although the relevant ligament structures were reconstructed, they still could not completely and realistically simulate the physiological state of the human body, which to a certain extent affected the accuracy of the test results. The influence of muscle tissue on the movement of the upper cervical spine in practical applications was ignored. Secondly, the finite element analysis simulated only the ROM and stresses of the atlantoaxial model after force was applied to demonstrate its feasibility and safety. The fatigue characteristics of internal fixation and the fatigue and fracture tests of internal fixation devices were neglected. A new LMSP system will need to be implanted in a cadaver model to further validate its safety. In subsequent experiments, the device will also be implanted into animal models to verify its feasibility in the healing of atlas fractures. Finally, we found that after posterior fixation of the four-part fracture model, the anterior arch of the atlas was still in a floating state, which could only simulate the state immediately after reduction and fixation but not after healing of the anterior arch fracture. Therefore, for the finite element study of ORIF treatment of unstable atlas fractures, creating a two-part fracture model involving the unilateral anterior and posterior arches may be more realistic.

## Conclusions

Both the LMSP and PSR systems can preserve the motion function of the upper cervical spine, particularly the rotational function of the atlantoaxial joint, and restore the stability of the upper cervical spine by a separate posterior approach surgery. The results of our FEM indicate that compared with the PSR system, the new atlas LMSP system may provide similar biomechanical stability in flexion, extension, and lateral bending but potentially higher stability in axial rotation. The LMSP system presents a theoretical basis for guiding clinical decision-making and shows promising prospects for future implementation.

## Data Availability

The data generated or analyzed in this study are included in the published article.

## Abbreviations

LMSP: Lateral mass screw–plate
PSR: Posterior screw and rod
FEM: Finite element model
ROM: Range of motion
MVMS: Maximum von Mises stress
ORIF: Open reduction and internal fixation
